# Picture worth a thousand words: Updating repeat photography for 21st century ecologists

**DOI:** 10.1002/ece3.7001

**Published:** 2020-11-09

**Authors:** William M. Hammond, Marie E. B. Stone, Paul A. Stone

**Affiliations:** ^1^ Department of Plant Biology, Ecology, and Evolution Oklahoma State University Stillwater OK 74078 USA; ^2^ Department of Biology University of Central Oklahoma Edmond OK 73034 USA

**Keywords:** digital photography, landscape disturbance, landscape ecology, Peloncillo Mountains, repeat photography

## Abstract

1. Anthropogenic climate change is altering every ecosystem on Earth. Understanding these changes requires quality baseline measurements of ecosystem states. While satellite imagery provides a coarse baseline for regional‐scale changes in vegetation, landscape‐scale observations are lacking. Ground‐based repeat photographic points (RPP) can provide this finer baseline. As precise visual records of ecosystems at a particular time, RPP provide rich data for diverse uses. Current methodology for establishing RPP, developed in the era of film cameras, requires placement of permanent markers in a landscape to provide accurate repeats over time. Another form of RPP involves relocating sites of historic photographs, to assess change between historic and present‐day photographs. Through a three‐year field survey, we synthesized these techniques to modernize repeat photography for the 21st century ecologist.
2. We established 100 RPP in the Peloncillo Mountains of New Mexico, recapturing 86 RPP in the three years (2015–2017) of the study. During our study, a large (>16,000 ha) complex of wildfires burned more than half of the RPP sites we established in the prior month, providing a unique opportunity to assess method accuracy after dramatic landscape disturbance by comparing burned, unburned, pre‐, and post‐fire RPP image recapture precision.
3. Our method produced 92% mean similarity for 86 RPP between original and repeated photographs, with no difference between burned and unburned sites. Interval between photographs did not cause a decline in similarity.
4. Our updated methods can be practically applied to nearly all terrestrial study systems. Landscape changes driven by human (e.g., effects of anthropogenic climate change, land use) and natural activities (e.g., wildfires, phenology, and hydrologic events) are especially well suited to our updated methods. Modern smartphones include the technology necessary (e.g., camera, GPS, and compass) to employ our method and provide a means for low‐cost deployment of the technique in diverse landscapes. We encourage broad adoption of this technique to establish baseline RPP of ecosystems across the globe, and the formation of a centralized database for repeat photography.

## Graphical abstract

Ecosystems are changing at an unprecedented pace due to anthropogenic change. Photographs can capture ecosystem states at a precise time, and by repeat photographs can be compared to quantify change. Here, we developed and field‐tested an updated method for repeat photography, finding high similarity using portable digital cameras without permanent landscape installations.

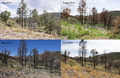

## 1 INTRODUCTION

**FIGURE 1:**
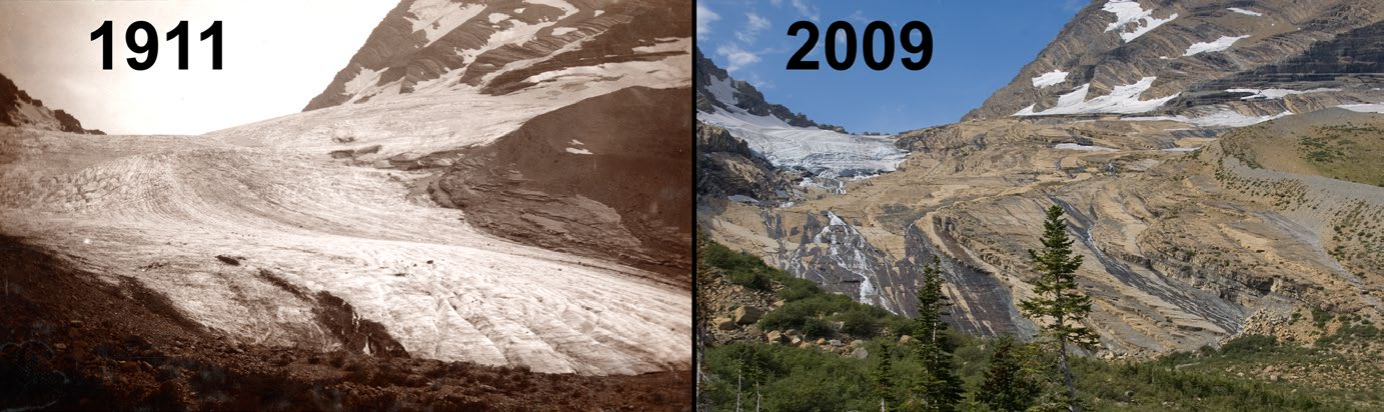
Repeat capture of historical photographs has long been used to document landscape change. Here, glacial retreat was documented in the near century‐long interval between the 1911 photograph (left) and the modern retake in 2009 (right). During the interval, the loss of ice and subsequent establishment of forest vegetation is readily apparent [Photos courtesy of Lisa McKeon (USGS Northern Rocky Mountain Science Center). Photo credit: 1911 Morton Elrond (Ross Toole Archives, U. of Montana); 2009 Lisa McKeon (USGS)]

Climate change is predicted to alter every ecosystem on Earth (IPCC, 2014). To understand fully the impact of climate change on ecosystems, a quality baseline must be established to measure future change. While satellite imagery provides a means to document baseline conditions for regional to global‐scale changes in vegetation, finer scale baselines are also needed. Indeed, recent meta‐analyses have found that most ecological studies operate at either <1 m^2^ or >1 km^2^ spatial scale, resulting in an observational gulf of scale at the landscape level (Estes et al., 2018). Ground‐based photographic collection points, hereafter referred to as Repeat Photo Points (RPP), provide this finer baseline at the landscape scale (as in Figure 1, see also Cerney, 2010; Webb, 2010). Repeat Photo Points provide precise visual records of ecosystems at a moment in time (Rogers et al., 1984; Smith et al., 2001).

By repeat collection of the same photographic point, change can be identified and measured. Examples of diverse changes measured at the landscape scale by repeat photographic studies include erosion and landslides (Khattak et al., 2010; Munroe et al., 2008), vegetation (Clark & Hardegree, 2005; Hendrick & Copenheaver, 2009; Masubelele et al., 2013, 2015; Zier & Baker, 2006), urbanization (Kull, 2005), rangeland and forest management (Boyd & Svejcar, 2005; Niraula et al., 2013), effects of land use and land cover change (Bass, 2011; Griffin et al., 2005; Kull, 2005) including cultural landscape change (Bass, 2011; Nüsser, 2001), ecological restoration (Stevenson, 1993), and glacial retreat (Byers, 2007, 2017; Masiokas et al., 2008; Moseley, 2006).

### 1.1 Repeat photography: fixed‐point photographs

Current methods for establishing and recapturing “fixed‐point” RPP involve inserting one permanent marker in view of the camera and another permanent marker at the location of the camera (Hall, 2001, 2002; Van Horn & Van Horn, 1996). Both markers are left in the environment as reference points between intervals of RPP capture (Van Horn & Van Horn, 1996). Markers assist in relocating RPP and ensure precise matches between original and repeated photographs. Compass bearings for camera directions are recorded separately, and field notes are recorded to promote relocation of the site. Additionally, notes are made regarding the camera model, lens used, and settings at which the image was captured (e.g., focal length, exposure). More recently, coordinates of sites have been recorded using global positioning system (GPS) devices. A physical copy of the original photograph is used during recapture to ensure that the angle matches that of the original photograph (Rogers et al., 1984). Studies using this method of RPP have produced consistent results (Hall, 2002). Using these existing methods, RPP have been established and monitored for the purpose of answering specific research questions (Webb, 2010). Recent advances in the fixed photograph point technique include long‐term “Phenocam” systems, where a digital camera is permanently installed for timeseries imaging of the same landscape (Brown et al., 2016). However, this method is restricted to the availability of infrastructure (e.g., electricity, Internet) and funding required to install and maintain such systems.

### 1.2 Repeat photography: historic recaptures

Despite the accuracy of using permanent markers, the widest application of repeat photography for scientific research has involved recapture of historic photographs rather than establishment of new RPP (Cerney, 2010; Hendrick & Copenheaver, 2009; Kull, 2005; Roush et al., 2007). Historic archives are searched for old, site‐specific, high‐quality photographs that contain landmarks or locale information to promote resighting of particular areas (Hendrick & Copenheaver, 2009; Roush et al., 2007; Webb, 2010). Historic photographs allow immediate review and assessment of change across a known time interval. The largest collection of these “historic recapture” repeat photographs is archived in the Desert Laboratory Repeat Photography Collection, maintained by the United States Geologic Survey (Webb et al., 2007). Historic photographs are limited in their contribution as baseline data in several ways. First, historic images are inherently encumbered with the bias of the original photographer. Second, historic images present a challenge to relocate the original photographic point in space, which can take considerable time. While historic photograph retakes are not exact repeats of the same image (as the standard method, with permanent markers provides), they have provided invaluable insight into what landscape changes have occurred in global ecosystems during the last century (Masubelele et al., 2014, 2015; Rogers et al., 1984; Webb, 2010). Finally, accurately recapturing a historic photograph also requires replicating the unknown weather (e.g., time of day, cloud cover) and camera (e.g., lens, tripod height) conditions to aid in discrimination between artifacts (e.g., shadows) and actual landscape change.

### 1.3 21st Century repeat photography: methods synthesis

**FIGURE 2:**
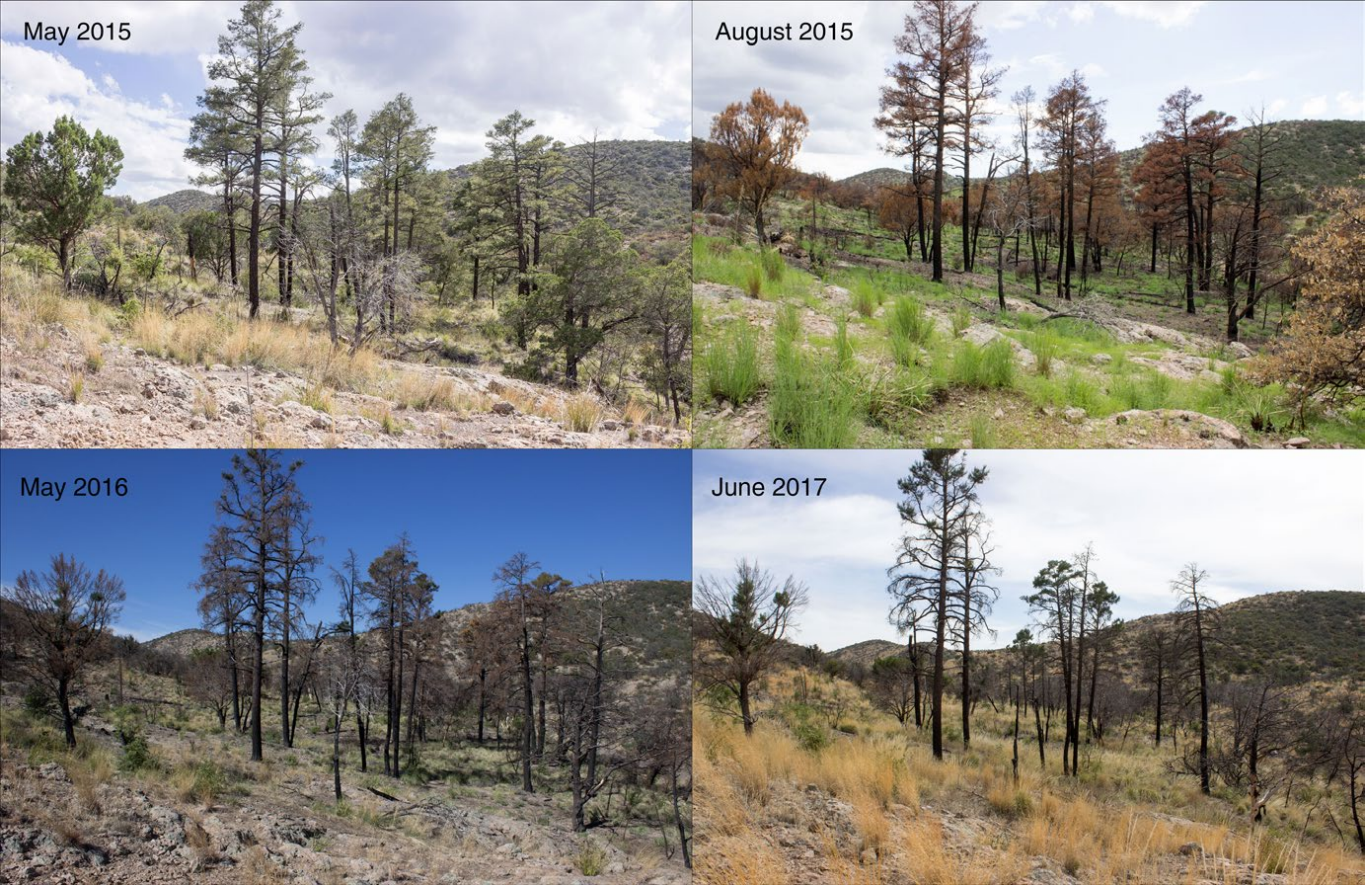
Repeat photographs of a stand of Chihuahua pine (*Pinus leiophylla*) in the Peloncillo mountains of New Mexico. This repeat photographic point was established in May 2015. The Guadalupe fire burned in late June 2015, just before the first recapture in August 2015. In August 2015, many trees have browning canopies. By May 2016, many trees were dead, and by June 2017 the stand of trees has significantly thinned

Synthesis of fixed‐point and historic recapture RPP methods would provide a powerful tool for evaluation of temporal change of landscapes and provide a means to fill the gulf of spatial scale representation in many disciplines of ecology (Estes et al., 2018). Ideal synthetic methods would produce matched photographs that are highly similar, without requiring relocation effort required using the historic photograph technique. Furthermore, the updated method should allow comparison of RPP without leaving permanent markers in the field, allowing addition of RPP to ongoing field research projects with little investment (Figure 2). Our study focused on testing the efficacy of modern equipment and synthesized methods in the absence of permanent landscape markers. In the Peloncillo Mountains of New Mexico, USA, we conducted a three‐year study using a historic photograph collection, along with establishing new RPP. During our study, wildfire burned half of our repeat photograph points, providing an opportunity to investigate our method's precision in both the presence and absence of dramatic landscape disturbance. We developed a comparison technique using digital image analysis to evaluate the effectiveness of our new method across time and disturbance.

## 2 MATERIALS AND METHODS

### 2.1 Study area

**FIGURE 3:**
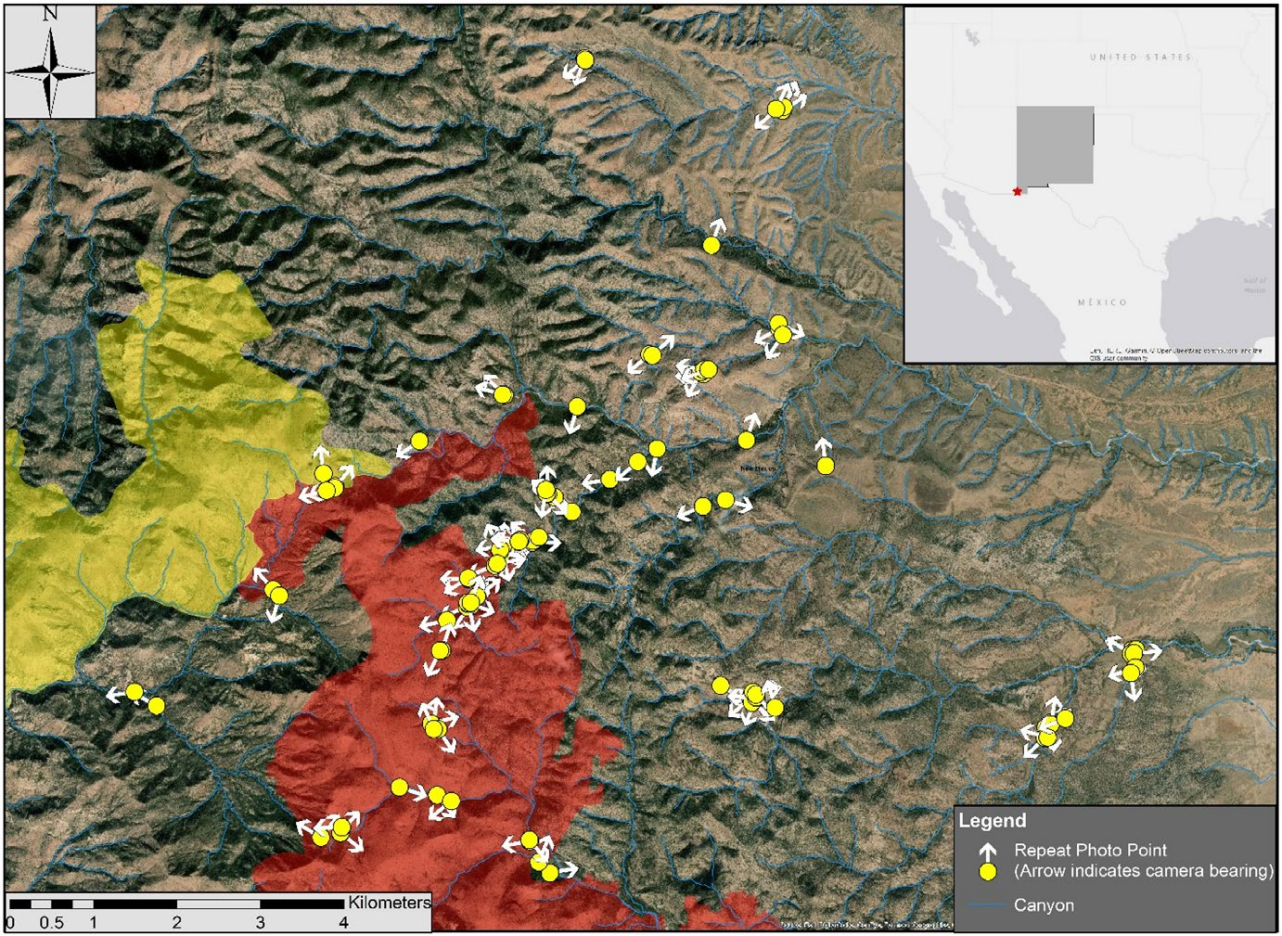
Repeat photographic points in the Peloncillo Mountains. Each yellow dot represents a single RPP (*n* = 100) and the white arrow indicates camera bearing of the photograph point. Inset map at top right illustrates the location of the study site in North America, with a red star. Shaded areas represent the burn perimeter for the Hog Canyon (yellow) and Guadalupe (red) fires

We tested the efficacy of our method for establishing and recapturing RPP in the Peloncillo Mountains of southwestern New Mexico (31.526 N, −108.977 W, Figure 3), a Madrean Sky Island range with a maximum elevation of ~6,000 m (Bodner et al., 2006). Climate in the region is seasonal and dry, typical of the Chihuahuan Desert, including distinct dry (April–June) and wet (July–September) seasons with high annual variation in precipitation, variable winter precipitation, and frequent drought (Bodner et al., 2006; Neilson, 1986). An ecological research project ongoing since 1994 at our study site (Stone, 2001; Stone et al., 2014) provided the opportunity to recapture historical photographs and establish new RPP.

### 2.2 Study aims

Our goal was to establish 100 RPP in the Peloncillo Mountains between May and August 2015. Additionally, we aimed to recapture a subset of the 2015 RPP in 2016 and 2017. Finally, we sought to relocate and recapture 15 historic field photographs (taken 1–16 years prior) of the site dating from the late 1990s and early 2000s. Original images were taken with several different cameras utilizing both film and digital technologies, replicating the challenges typically encountered with relocating and recapturing historical photographs, as described above.

### 2.3 Equipment

We used the same camera (Canon T3i D‐SLR, 18–55 mm lens) and field equipment for both establishment of novel RPP and recapture of RPP (including those based on historical, film‐based photographs). We mounted the camera on a portable tripod and inserted a leveling cube to level the camera. We used a metric measuring tape to record the distance (in cm) from the camera lens to the ground and a Silva model 515 field compass to record the magnetic bearing of the camera lens. We recorded the coordinates of each RPP using a handheld GPS unit (±3 m, Garmin eTrex Vista CX GPS). We recorded the filename of the photograph, as all relevant camera information (model, resolution, focal length of lens used, camera settings during capture) was automatically imbedded in the digital photograph by the camera.

### 2.4 Recapture methods

Recapturing RPP first involved relocating the RPP using a GPS. With an error of ±3 m, arriving at the GPS location merely gets one in the neighborhood. Before framing, we used a tripod and measuring tape to match the camera height recorded at establishment of the RPP. The bearing was then sighted with the field compass to align with the camera's bearing in the previous photograph. We used a printed copy (10 cm × 15 cm) of the previous photograph that was referenced while framing the shot. With the camera already aligned to the original bearing, remaining error in position was corrected by physically moving the tripod in space until the shot was appropriately framed.

### 2.5 Recaptures and Wildfires

In total, we established 100 RPP and recaptured 86 RPP (including 15 based on historic photographs) during the study. During May 2015, 50 RPP were established in the Peloncillo Mountains, including RPP (*n* = 15) anchored on historic field photographs. Before returning in August 2015 to complete the final 50 RPP, two wildfires (Hog Canyon and Guadalupe fires) burned over 16,000 ha of the Peloncillo Mountains. In August 2015, 50 additional RPP were established with the majority being established in recently burned areas (Figures 2 and 3). In May 2016, we returned to recapture RPP (*n* = 49), of which 29 had burned between establishment and recapture, and 20 had not burned. In May 2017, we recaptured RPP (*n* = 22) two years from establishment in 2015. Of the 22 recaptures taken two years after establishment, 11 had burned in 2015 and 11 had not burned during the wildfires of June 2015.

### 2.6 Image comparison methods

Once RPP were recaptured, percent similarity between the original (e.g., digitally established in 2015 or digitized historic field film) photographs and recaptures was calculated. We calculated percent similarity using GNU Image Manipulation Program (Kimball et al., 2014). Original and repeat photographs were layered atop one another, and the opacity of the top layer reduced to forty percent. This provided a transparent overlay, allowing image alignment by moving the top layer to match the bottom layer. Once alignment was complete, we cropped images to include all overlap (shared pixels) between the images. We divided the number of pixels composing the cropped images by the number of pixels in the uncropped images to calculate percent similarity for each image using the equation:

$$\left[\left(\frac{\mathrm{pixels}\mathrm{count}\mathrm{of}\mathrm{cropped}\mathrm{image}}{\mathrm{pixel}\mathrm{count}\mathrm{of}\mathrm{original}\mathrm{image}}\right)\times 100\right]=\%\mathrm{similarity}$$

We used ANOVA to test for a difference in mean percent similarity between burned, unburned, and historic repeat photographs, and a Tukey post hoc test to determine which were significantly different. Additionally, we tested for differences in percent similarity between burned and unburned RPP sites using a *t* test. Finally, we used a linear model in program R (version 3.6.2) to determine whether there was an effect of camera bearing on percent similarity.

## 3 RESULTS

### 3.1 Method precision

**FIGURE 4:**
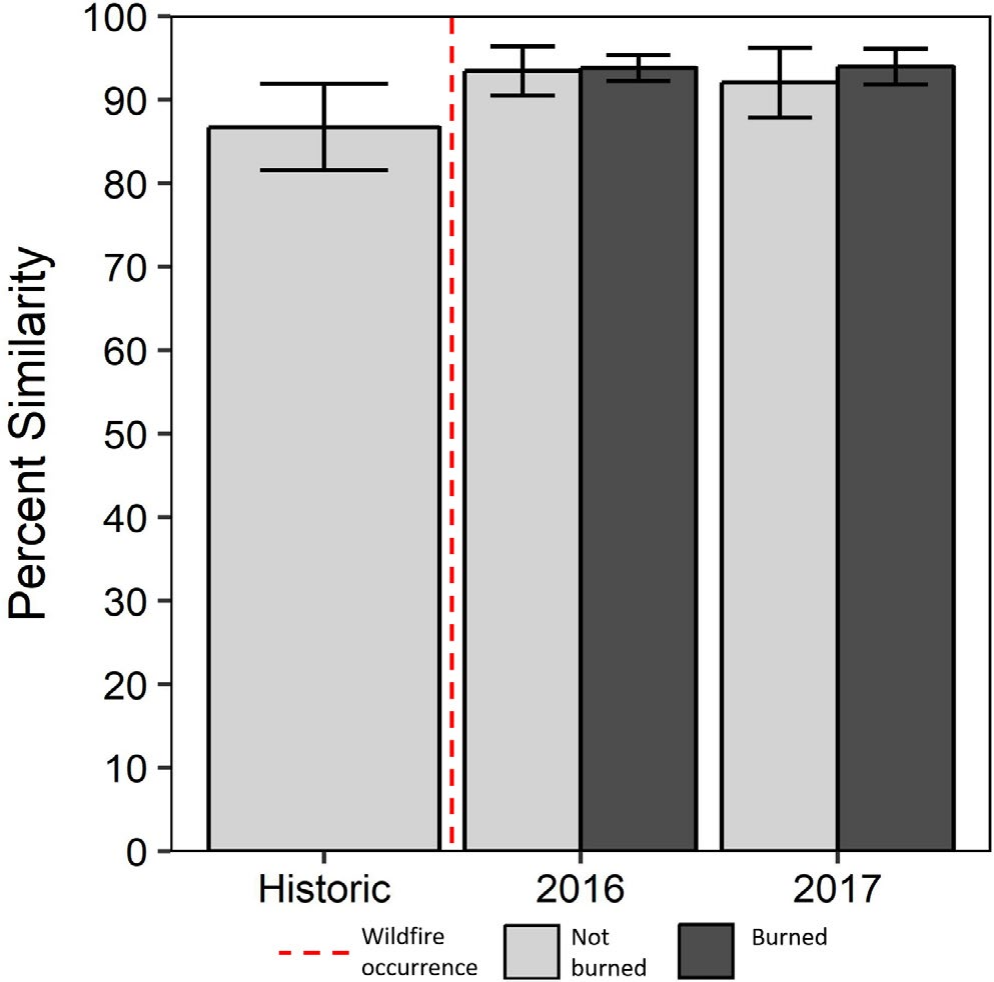
Bar height indicates mean percent similarity for RPP recaptured in 2015, 2016, and 2017. Light gray bars are RPP sites that did not burn during the 2016 fire season, while dark gray bars are RPP sites that burned (error bars = 95% CI). Historic photographs were recaptured in 2015 (*n* = 15) and aligned for percent similarity with digital versions of the original historic photographs, while 2016 (*n* = 49) and 2017 (*n* = 22) recaptures were aligned to RPP taken during establishment in 2015

We observed a 92.3 (±6.4%) mean percent similarity for the 86 RPP recaptured during our study. Of these, the average percent similarity for RPP established on historic photographs (*n* = 15) was 86.7% (±9.4%, Figure 4). The interval between capture of historic photographs and recapture in 2015 ranged from 1 to 16 years, with no apparent trend for percent similarity as the interval increased (Table 1). For RPP established in 2015, recaptures taken after one year (*n* = 49) were 93.7% (±5.0% *SD*) similar, and recaptures taken after two years (*n* = 22) were 93.2% (±4.4% *SD*, Figure 4) similar. Percent similarity was significantly lower for RPP established on historic photographs than for RPP established and recaptured exclusively with our method (ANOVA, *F* = 8.368, *p* < .001, Tukey HSD *p* < .01 for 2016:Historic & 2017:Historic).

**TABLE 1:** Percent similarity for the 15 RPP based on historic photographs, grouped by the interval, or number of years between the initial photograph and recapture in 2015

RPP interval (years)	*n*	Similar %	*SD*
1	4	88.7	7.7
2	1	66.1	
3	2	83.1	9.1
6	1	73.9	
8	3	89.4	7.5
9	1	97.4	
11	1	93.4	
16	2	90.5	5.1

Note

Overall percent similarity was 86.7%. For each interval, the number of repeat photographs represented (*n*) is listed, and for intervals with more than one RPP, the listed percent similar is a mean value (with accompanying standard deviation).

**FIGURE 5:**
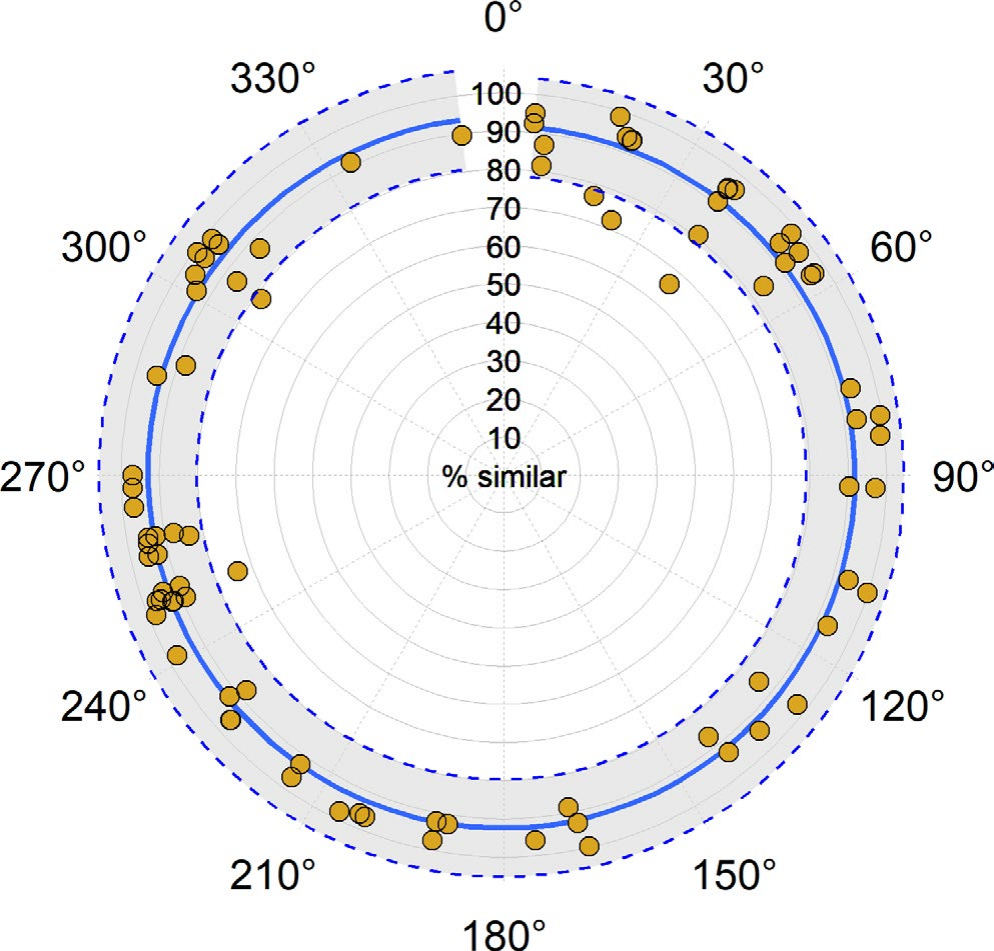
Linear regression between percent similarity and camera bearing plotted in polar coordinates. The solid blue line is the model fit for percent similarity, while dashed blue lines indicate the upper and lower 95% confidence interval, with gray shading between. Yellow circles represent the percent similarity of an individual RPP (*n* = 86) from the Peloncillo Mountains. Regardless of bearing, model fit was for >90% similarity. The lowest bound of the confidence interval was approximately 80% similar across all bearings

While bearing of RPP was guided by the landscape at each point, our 86 retakes represented nearly all bearings (Figures 3 and 5). A linear regression of camera bearing and percent similarity was not significant (*p* = .30, *R* ^2^ = .001). Across all bearings, model fit was for percent similarity to be >90% (Figure 5).

### 3.2 Effect of wildfire

Unburned RPP (*n* = 41) averaged 90.7 (±8.1%) similarity, while burned RPP (*n* = 45) averaged 93.8 (±3.7%). No difference was observed between burned and unburned RPP recaptures (*t* test, *p* = .494, Figure 4). We documented extensive vegetation mortality in response to wildfire (Figure 2), but effects were different for open grown communities of low‐stature plants and closed canopies of taller trees where fires appear to have burned more intensely. Two months after the wildfires, we observed grasses, forbs, and low‐stature woody species vigorously resprouting (Figure 2b). In contrast, we documented stand‐level mortality of *Pinus leiophylla* (RPP35, RPP72, and RPP74) and *Juinperus deppeana* (RPP58, RPP59, RPP60, RPP61, and RPP68), both of which are long‐lived fire‐adapted trees species.

### 3.3 Observed landscape changes

**FIGURE 6:**
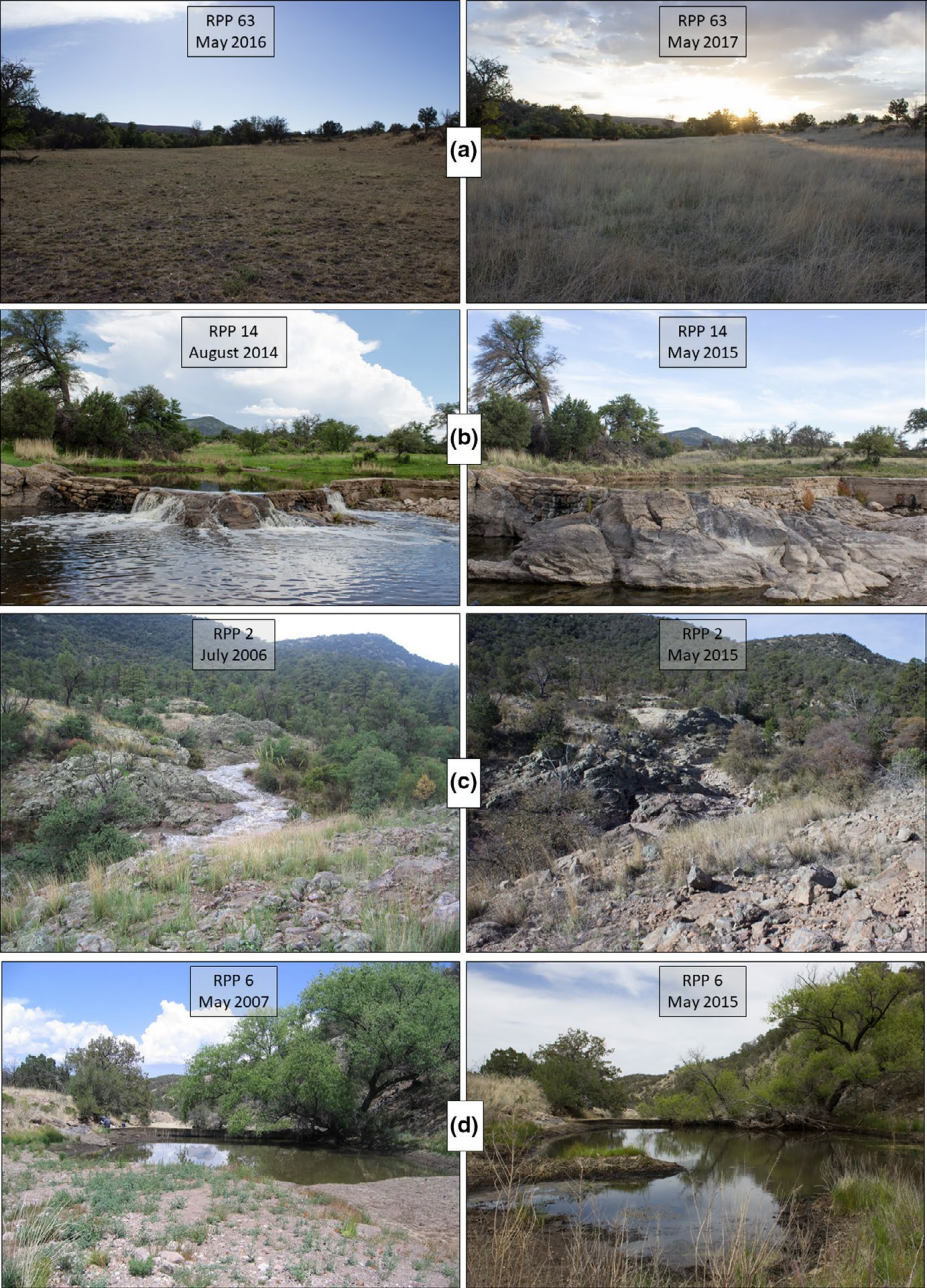
Composite of RPPs representative of observed changes. Historic or initial RPP images are on the left and recaptures on the right. In panel A, the impact of grazing cattle was documented in a landscape dominated by grasses and forbs. Panels B and C show variation in hydrologic regimes in manmade and natural systems, respectively. Panel D shows the impact of a restoration effort to remove silt from a tank and restore wildlife habitat

We observed numerous other qualitative changes in the landscape between establishment and recapture, not related to the wildfire. Landscape use effects from grazing of livestock (Figure 6a), changes in phenology of plants, and fluxes in water availability in canyons and tanks (Figure 6b,c). In RPP6 (Figure 6d), we observed the impact of habitat restoration. The historic photograph shows silt accumulation after a wildfire, whereas the recapture shows the same habitat three years after silt removal (Stone et al., 2014).

## 4 DISCUSSION

We tested a method that modernizes the establishment and recapture of RPP using technology available in modern cameras and smartphones. We found a 93.7% similarity between original and recapture RPP, demonstrating our method is an effective means of documenting change through photography. Our method is simple—any person can record a GPS location, height of camera lens from ground, and compass bearing. The remaining relevant information is stored in the digital photographic file. This allows for wide‐scale adoption of our method.

In the Peloncillo Mountains, as in much of the American Southwest, anthropogenic climate change is predicted to elevate average temperatures, accompanied by increased frequency, intensity, and duration of drought (Cook et al., 2015; Seager et al., 2013). Already, these dramatic changes have led to observations of widespread vegetation mortality (IPCC, 2014; McDowell et al., 2011). Effects thus far have appeared as desertification, with grasslands turning into shrublands in much of the southwest since the 1850s (Buffington & Herbel, 1965). Modeling of processes driving landscape‐level changes in the Southwest suggests that profound disturbance of water and nutrient distributions has occurred in the area, and continuing landscape‐level change is expected (Mueller et al., 2007). Thus, documenting baseline states of these ecosystems represents an urgent need. Impacts of global climate change are not limited to the American Southwest, however, and our updated RPP methods allow documentation, an opportunity for analysis, and an insight of ecosystems change across space and time not available in near or remote‐sensing platforms presently available to ecologists.

Encouraging adoption of our method on a wide‐scale demands that precision in repeats be attainable regardless of the technology utilized. RPP’s we established on historic photographs (*n* = 15) demonstrated precision when different initial camera equipment was used, across 16 years. While novel RPP and their subsequent recaptures were conducted with the same camera, the historic photographs we recaptured originated from 35mm film cameras and three early models of digital cameras. While percent similarity was slightly lower for these recaptures, the overall similarity (86.7%) provides ample overlap between the historic and repeat photographs through which changes may be assessed. Demonstrating precision across multiple media and technological iterations reinforces the efficacy of our method through time, during which future technological changes in photography (e.g., cameras, media format) are likely to occur.

Withstanding long intervals between RPP recaptures is another crucial characteristic of precision in retakes of RPP. We expected percent similarity decrease with time since recapture, as landscape‐level changes in plant communities and effects of erosion should accumulate with increasing interval between capture of RPP. Our study showed that with an interval of up to 16 years for our historic photographs, we observed a high percent similarity.

One of the challenges encountered during the establishment of RPP is the inherent human bias when taking photographs (Hendrick & Copenheaver, 2009; Zier & Baker, 2006). Our RPP were not selected at random locations, or in random directions. Instead, a set of points that sampled diverse landscapes was selected for this study. It is reasonable to assume that those establishing RPP will take a similar approach in selecting locations that interest them (Rogers et al., 1984). By offsetting inherent bias in establishment of RPP, recapture allows visualization of all changes that have occurred since the last capture, whether they were intended to be measured or not. Using RPP data, both intended and unintended information is captured, which can allow the testing of diverse anticipated and unanticipated hypotheses. A clear example of this is evident in Figure 1, where our long‐term RPP established in 2015 experienced a significant, high‐intensity wildfire that killed century‐old trees. While we had no means of knowing this stand would burn, having the RPP established positioned us to investigate initial and lasting impacts from the wildfire.

The Desert Laboratory Collection pioneered centralization of repeat photography collections, and established standards for the curation of repeat photographic data in the era of film cameras (Webb, 2010). The next step in this technology is to connect RPP being recorded globally into a central, easily accessible, and open‐source online repository, a Repeat Photographic Point Repository (RPPR). By making existing RPP available to everyone, recaptures of RPP may be taken by anyone. An example of engaging citizen scientists in repeat photography can be seen at the rePhotoSA, the repeat photography project of southern African landscapes (http://rephotosa.adu.org.za) website, where contribution of both historic photographs and their recapture is documented for much of southern Africa. The database should include necessary information to locate RPP, a copy of the photograph taken at establishment, and the ability to post recaptures to the RPPR with necessary data attached. It is technologically simple to imbed in each RPP image all information necessary for recapture. Recording these data within the photograph file promotes integrity of the system by preventing disjunction between photographs and metadata. Security of RPP data would involve duplicate copies of all RPP stored on servers in separate locations. The database should be made available in as many languages as possible, encouraging global participation in collection of baseline RPP against which global change may be measured at the landscape scale—an often‐underrepresented scale of observation in the field of ecology (Estes et al., 2018). As regional efforts to document landscape change with repeat photography are already underway (e.g., as with rePhotoSA, above), partnership with regional experts and entities will be essential in assembling a truly global database of RPP data. Our future plans include development of the RPPR, which will require identifying these international collaborators, securing funding, and providing a long‐term data curation best practices, ultimately providing a central location for the storage and use of RPP for scientific research.

We encourage all field researchers to begin incorporating this method when they take photographs in the field. As each RPP is established, the amount of baseline data upon which future changes can be measured will grow. At the turn of the twentieth century, the maxim “a picture is worth a thousand words” appeared as photographs conveyed complex information with a high level of detail not previously available in printed media. If a picture is worth a thousand words, what would thousands of pictures documenting current baseline states of ecosystems across the globe be worth to researchers?

## CONFLICT OF INTEREST

None declared.

## AUTHOR CONTRIBUTION

**William M. Hammond:** Conceptualization (equal); Data curation (lead); Formal analysis (equal); Investigation (supporting); Methodology (supporting); Visualization (lead); Writing‐original draft (lead); Writing‐review & editing (equal). **Marie E. B. Stone:** Investigation (equal); Methodology (equal); Writing‐review & editing (equal). **Paul A. Stone:** Conceptualization (equal); Formal analysis (equal); Investigation (equal); Methodology (equal); Writing‐original draft (equal); Writing‐review & editing (equal).

## Data Availability

All image data are available in a Figshare repository (https://doi.org/10.6084/m9.figshare.c.4987655.v2).
